# Catalytic Analysis of APOBEC3G Involving Real-Time NMR Spectroscopy Reveals Nucleic Acid Determinants for Deamination

**DOI:** 10.1371/journal.pone.0124142

**Published:** 2015-04-13

**Authors:** Keisuke Kamba, Takashi Nagata, Masato Katahira

**Affiliations:** 1 Graduate School of Energy Science, Kyoto University, Gokasho, Uji, Kyoto, Japan; 2 Institute of Advanced Energy, Kyoto University, Gokasho, Uji, Kyoto, Japan; Centro de Biología Molecular Severo Ochoa (CSIC-UAM), SPAIN

## Abstract

APOBEC3G (A3G) is a single-stranded DNA-specific cytidine deaminase that preferentially converts cytidine to uridine at the third position of triplet cytosine (CCC) hotspots. A3G restricts the infectivity of viruses, such as HIV-1, by targeting CCC hotspots scattered through minus DNA strands, reverse-transcribed from genomic RNA. Previously, we developed a real-time NMR method and elucidated the origin of the 3'→5' polarity of deamination of DNA by the C-terminal domain of A3G (CD2), which is a phenomenon by which a hotspot located closer to the 5'-end is deaminated more effectively than one less close to the 5'-end, through quantitative analysis involving nonspecific binding to and sliding along DNA. In the present study we applied the real-time NMR method to analyze the catalytic activity of CD2 toward DNA oligonucleotides containing a nucleotide analog at a single or multiple positions. Analyses revealed the importance of the sugar and base moieties throughout the consecutive 5 nucleotides, the CCC hotspot being positioned at the center. It was also shown that the sugar or base moieties of the nucleotides outside this 5 nucleotide recognition sequence are also relevant as to CD2's activity. Analyses involving DNA oligonucleotides having two CCC hotspots linked by a long sequence of either deoxyribonucleotides, ribonucleotides or abasic deoxyribonucleotides suggested that the phosphate backbone is required for CD2 to slide along the DNA strand and to exert the 3'→5' polarity. Examination of the effects of different salt concentrations on the 3'→5' polarity indicated that the higher the salt concentration, the less prominent the 3'→5' polarity. This is most likely the result of alleviation of sliding due to a decrease in the affinity of CD2 with the phosphate backbone at high salt concentrations. We also investigated the reactivity of substrates containing 5-methylcytidine (*5mC*) or 5-hydroxymethylcytidine, and found that A3G exhibited low activity toward *5mC*.

## Introduction

Human apolipoprotein B mRNA-editing enzyme-catalytic polypeptide-like 3G (APOBEC3G or A3G) is a single-stranded DNA (ssDNA)-specific cytidine deaminase that converts cytidine (C) to uridine (U) [1–6]. A3G possesses two consensus zinc-finger-type cytidine deaminase motifs [7], of which only the C-terminal one (CD2) is catalytically active [8, 9]. As for the sequence specificity of the deaminase activity, the triplet cytosine (CCC) in ssDNA is called a 'CCC hotspot' since the third cytidine of CCC (underlined) is most effectively deaminated by A3G [1–6, 10–12], while the second cytidine of dicytidine (CC) is also targeted but rather less effectively [13]. A3G targets the newly synthesized minus strand of the human immunodeficiency virus 1 (HIV-1) and thus introduces a significant level of mutations into the viral genome, resulting in disruption of the infectivity of virus-infectivity-factor (Vif)-deficient HIV-1 strains [1–3, 6, 7, 14–19]. The molecular structure of free A3G CD2 was determined previously [11, 20, 21], however, that of the A3G:ssDNA complex remains elusive.

DNA-binding of A3G has been analyzed by various methods, including electrophoretic mobility shift assays (EMSA) [10, 21, 22], steady-state rotational-anisotropy binding assays [10], single-molecule fluorescence resonance energy transfer (sm-FRET) [23], and atomic force microscopy (AFM) [24, 25], by which it was found that A3G binds to ssDNA in a sequence-nonspecific manner and slides along the ssDNA without directional preference. A uracil-DNA glycosylase-based assay (UDG-based assay) has been popular for analysis of the deaminase activity of A3G [5, 10–13, 26]. UDG-based assays revealed that A3G deaminates CCC hotspots in a location-dependent manner, known as 3′→5′ polarity, which is a phenomenon by which a CCC hotspot that is located close to the 5' end is deaminated more effectively than one that is less close to the 5′ end [10]. This phenomenon was originally observed *in vivo* as a 5′ to 3′ gradient of G to A hyper-mutations in HIV-1 RNA, which is transcribed from minus strand DNA [4, 5].

We previously utilized real-time NMR spectroscopy to monitor the deamination reaction, with which one can detect the reaction directly in a site-specific manner with high temporal and spatial resolution [11]. The advantage of this method is that it is sensitive as to weak interactions because highly concentrated ssDNA (100 μM order) can be used. Recently, this method has become increasingly popular and has been used by other groups [12, 27]. Our real-time NMR method had revealed that A3G deaminates the third cytidine of a CCC hotspot much faster than the second one (CCC) [11]. In the subsequent study, we monitored the deamination reactions of ssDNA containing two CCC hotspots and revealed that A3G CD2 can deaminate the two hotspots with 3′→5′ polarity. Furthermore, by construction of a kinetic model, in which nonspecific protein:ssDNA binding and sliding processes are incorporated, we quantitatively analyzed the 3′→5′ polarity of deamination by A3G CD2 [28]. It was revealed that the 3′→5′ polarity of A3G can be rationally explained by introducing the sliding direction-dependent catalytic rate. The analysis provided the values for various kinetic parameters, and importantly the catalytic rate of A3G CD2 was shown to be higher (ca. five-fold) when A3G CD2 approaches the target cytidine in the 3' to 5' direction rather than in the opposite direction.

Previously, substrate-binding features and the deamination specificity of A3G were analyzed by means of so-called nucleotide analog interference mapping [26]. With this method, ssDNA substrates containing single or multiple nucleoside analogs, that have a modification in the base, sugar or phosphate moiety, are used for the UDG-based assay. Introduction of 2′-O-methyl modification, methyl phosphotriester modification, or an abasic site at a single position in the substrate ssDNA revealed that the sugar and phosphate moieties of the nucleotides within the CCC hotspot and its vicinity are important for substrate recognition by A3G. Moreover, introduction of pyrimidine base analogs at positions adjacent to the deamination target site clearly showed that A3G dictates the exocyclic groups in pyrimidines 1–2 nt 5′ to the target cytosine, therefore the authors concluded that the base moieties of these nucleotides are the most critical for A3G to recognize the target cytidine.

Here, we applied the real-time NMR method to analyze the catalytic activity of A3G CD2 toward ssDNA oligonucleotides containing a nucleotide analog at a single or multiple positions to identify nucleic acid determinants for deamination. Then, long ssDNA oligonucleotides, each containing two CCC hotspots connected by a long linker sequence of either deoxyribonucleotides, ribonucleotides or abasic deoxyribonucleotides were used as substrates to identify the chemical moieties of nucleotides that play a key role in the sliding of A3G CD2. To confirm the importance of the identified chemical moieties, the dependency of the 3′→5′ polarity on the NaCl concentration was examined.

Among the DNA modifications, methylation of cytidine at its C-5 position is known as a major epigenetic mechanism linked to gene regulation in development and in tumorigenesis [29], while DNA demethylation is one of the crucial processes for genome reprogramming during early embryogenesis [29]. Several pathways for DNA demethylation have been suggested, one of which involves 5-methylcytidine (*5mC*) to 5-hydroxymethylcytidine (*5hmC*) conversion by Ten-eleven translocation (Tet) proteins [30], deamination of *5hmC* by several deaminases (AID and APOBEC family proteins) [31–33], and subsequent replacement of a 5-hydroxymethyluracil-guanine mispair by MBD4 or thymine-DNA glycosylase (TDG) [34]. Although AID and APOBEC family proteins were hypothesized to participate in the demethylation pathways via deamination of *5mC* and *5hmC*, AID and many of the APOBEC family proteins were shown to be poor enzymes for deamination of *5mC* and *5hmC* [35, 36]. Among the APOBEC family proteins, it was shown recently *in vitro*, using a DNA glycosylase-based assay, and *in vivo* that APOBEC3A exhibits deamination activity toward C and *5mC* [37, 38], but that A3G exhibits activity only toward C [37, 38]. In the current study, substrates containing *5mC* or *5hmC* at the deamination target site were subjected to real-time monitoring to investigate the activity of A3G toward them by direct observation.

## Materials and Methods

### Preparation of proteins and oligonucleotides

Recombinant A3G CD2 (residues 193–384) was expressed and purified as described previously [11]. All oligonucleotides including DNA oligonucleotides containing a nucleotide analog (ribonucleotide, abasic deoxyribonucleotide, 5-methylcytosine deoxyribonucleotide or 5-hydroxymethylcytosine deoxyribonucleotide) at a single or multiple positions (Table 1) were purchased from Fasmac Co., Ltd.

**Table 1:** Oligonucleotides used in this study.

Substrate Name	Sequence	Length
	12345678910	
s1_DNA	AAACCCGAAA	10
s1_A2r	AaACCCGAAA	10
s1_A3r	AAaCCCGAAA	10
s1_C4r	AAAcCCGAAA	10
s1_C5r	AAACcCGAAA	10
s1_C6r	AAACCcGAAA	10
s1_G7r	AAACCCgAAA	10
s1_A8r	AAACCCGaAA	10
s1_A9r	AAACCCGAaA	10
s1_RNA	aaacccgaaa	10
s1_D2-8/r	aAACCCGAaa	10
s1_D2-7/r	aAACCCGaaa	10
s1_D3-8/r	aaACCCGAaa	10
s1_D3-7/r	aaACCCGaaa	10
s1_D3-6/r	aaACCCgaaa	10
s1_D4-7/r	aaaCCCGaaa	10
s1_D2-8/X	XAACCCGAXX	10
s1_D2-7/X	XAACCCGXXX	10
s1_D3-8/X	XXACCCGAXX	10
s1_D3-7/X	XXACCCGXXX	10
s1_D3-6/X	XXACCCXXXX	10
s1_D4-7/X	XXXCCCGXXX	10
	6 34	
s2_DNA	AAACCCGAA_21_AACCCGTA_22_	58
s2_RNA	aAACCCGAa_21_AACCCGTA_22_	58
s2_Abasic	XAACCCGAX_21_AACCCGTA_22_	58
s3_DNA	AAACCCGAA_21_AACCCGTAA	38
s4_C	AAAACCGAAA	10
s4_*5mC*	AAAAC*5m*CGAAA	10
s4_T	AAAACTGAAA	10
s4_*5hmC*	AAAAC*5hmC*GAAA	10
s4_*5hmU*	AAAAC*5hmU*GAAA	10

Deoxyribonucleotides and ribonucleotides are presented in large and small letters, respectively. “X” denotes abasic deoxyribonucleotide. 5-methylcytidine, 5-hydroxymethylcytidine, and 5-hydroxymethyluridine are shown as *5mC*, *5hmC*, and *5hmU*, respectively.

### Deaminase assay involving the real-time NMR method

All NMR spectra were recorded at 25°C on a Bruker DRX600 spectrometer equipped with a cryogenic probe and a Z-gradient (Bruker Biospin). A final concentration of 200 μM each DNA substrate was dissolved in 20 mM Tris-HCl (pH 7.5), 30 mM NaCl, 10 μM ZnCl_2_, 5 mM DTT, and 5% deuterium oxide. A final concentration of 0.8, 2.0, 20.0, or 70.0 μM A3G CD2 was used. To examine the NaCl concentration-dependence of A3G CD2's deaminase activity, the NaCl concentration was set to 1, 5, 15, 30, 45, 55, 65, 100, 150, 200, 250, 300, 350, 400, or 500 mM. After the addition of A3G CD2, 2D TOCSY (mixing time, 20 ms) and 2D ^1^H–^13^C HSQC spectra of each DNA substrate were recorded at different time points to monitor the deamination reaction in real-time. Water signal suppression was achieved by 3-9-19 watergate and echo-antiecho pulse schemes in each 2D TOCSY and 2D ^1^H–^13^C HSQC measurement. Resonance assignments of the oligonucleotides were carried out using 2D TOCSY and 2D ^1^H–^13^C HSQC spectra [11, 28]. Spectra were processed with NMRPipe [39], and analyzed using SPARKY [40].

The intensities of the cytidine peaks were plotted against time. The data were fit to a single exponential decaying function, using the following equations, by which apparent deamination rate constants were derived:

$$I(t)=I_{0}exp(-kt)+I_{\infty }\;,$$ (1)

,where *I*_∞_ is the baseline of the spectrum, *I*_0_ + *I*_∞_ the initial peak intensity at time point zero obtained by extrapolation after plotting, and *k* the apparent deamination rate constant, all of which were obtained through linear least-squares analysis. The error bar was estimated as follows. Firstly, the error of the NMR signal intensity was obtained from the base level noise of each NMR spectrum. Then the error of the rate constant was calculated from data sets constructed by Monte Carlo simulation using the error of the NMR signal intensity. Finally, the errors of the relative activity and the 3′→5′ polarity was obtained by means of error propagation calculation. The value for the apparent deamination rate constant for each oligonucleotide was defined as an index of activity.

We defined the 3′→5′ polarity with the following equation:

$$3'\to 5'\text{polarity}=\frac{k_{\text{(C6)}}}{k_{\text{(C34)}}}\;,$$ (2)

,where *k*_(C6)_ and *k*_(C34)_ are the apparent deamination rate constants at positions C6 and C34 of 38- and 58-mer substrates, respectively (Table 1).

## Results and Discussion

### The effect of ribonucleotide substitution at a single position of a substrate ssDNA on A3G CD2's deaminase activity

**Fig 1:**
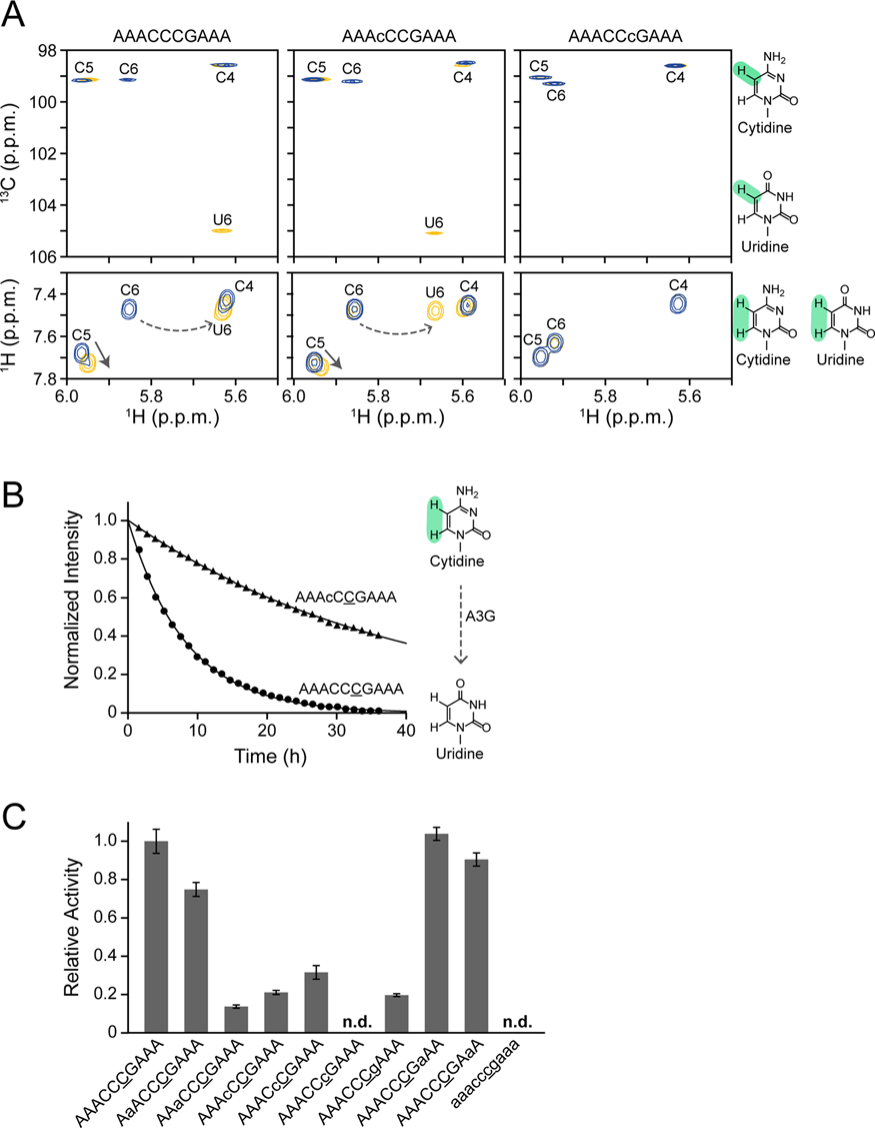
Real-time monitoring of deamination reactions for substrates containing a single ribonucleotide substitution. **(A, B)** Examples of real-time monitoring. ^1^H-^13^C HSQC spectra (A, top) and TOCSY spectra (A, bottom) of ssDNA substrates, AAACCCGAAA (s1_DNA), AAAcCCGAAA (s1_C4r), and AAACCcGAAA (s1_C6r) (c: cytosine ribonucleotide), in which C to U conversion occurs at underscored C6. Blue signals in ^1^H-^13^C HSQC spectra present before addition of A3G CD2, while yellow signals present 107 hours (s1_DNA), 74 hours (s1_C4r), and 90 hours (s1_C6r) after addition of A3G CD2. Blue and yellow signals in TOCSY spectra each present before and 36 hours after addition of A3G CD2. Signals of C6 and U6 are connected by a broken arrow, while a solid arrow indicates the signal perturbation of C5 due to the C6 to U6 conversion. The time course of the intensity change in TOCSY spectra was monitored for C6 (underscored) of AAACCCGAAA and AAAcCCGAAA (B). Structural formulas of cytosine and uracil are shown (highlighted positions, H5–C5 and H5–H6, were monitored). **(C)** Relative activity was defined as the ratio of the deamination rate constant, *k*, obtained for C6 of each substrate containing a single ribonucleotide substitution to that obtained for C6 of AAACCCGAAA.

The real-time NMR method was applied to monitor the deaminase activity of A3G CD2 toward a series of DNA oligonucleotides, each containing ribonucleotide substitution (more specifically, 2'-OH substitution) at a single position. The standard substrate was a 10-mer DNA oligonucleotide, AAACCCGAAA (s1_DNA in Table 1), the corresponding ribonucleotides at positions 2 to 9 in the other substrates each being substituted (s1_A2r to s1_A9r in Table 1). Fig 1A shows examples of real-time monitoring of the deamination reactions for three substrates, s1_DNA, s1_C4r and s1_C6r. The top and bottom panels of Fig 1A show TOCSY (H5–H6 correlation signals) and ^1^H-^13^C HSQC (H5–C5 correlation signals) spectra, respectively. The three blue signals in each spectrum in Fig 1A are those of C4, C5, and C6 of the CCC hotspots of the substrates before the addition of A3G CD2. Several hours after the addition of A3G CD2 (see legend to Fig 1A), the spectra of the different substrates showed different outcomes. In the cases of s1_DNA and s1_C4r, along with conversion of the target cytidine to uridine (C6 to U6), the signal of C6 disappeared and decreased, respectively, and the U6 signal appeared. On the other hand, for s1_C6r, in which the target cytidine is substituted by a ribonucleotide, no signal change was evident, indicating that deamination failed. Thus, it was clearly shown that the real-time NMR method can distinguish the reactivity for the different substrates.

For detailed analysis with the real-time NMR method, we chased the intensity change of the H5–H6 TOCSY correlation signal with the targeted cytidine, C6, in each substrate. Fig 1B presents the results obtained for s1_DNA and s1_C4r as examples. It is clear that the decrease in the C6 intensity is only slight in the case of s1_C4r. This indicates that ribonucleotide substitution (2'-OH substitution) at the fourth position, that corresponds to the first cytidine of a CCC hotspot, negatively affects A3G CD2's activity. We performed this analysis for the other substrates listed in Table 1, from s1_DNA to s1_RNA, and a deamination rate constant, which can be considered as an index of activity, was calculated. Relative activity was also calculated for further quantitative analysis.

Fig 1C compares the relative activities of A3G CD2 toward ssDNA substrates, each having a single ribonucleotide substitution at different positions. The relative activity of A3G CD2 toward RNA substrate (s1_RNA) is also included as a negative control. A3G CD2 failed to deaminate this substrate as well as s1_C6r, the latter being mentioned above, and in both cases a 2'-OH substitution was introduced at the targeted cytidine. Fig 1C indicates that the negative effect of a 2'-OH substitution on reactivity is great when the substitution is introduced within a CCC hotspot, and also at the positions immediately flanking the 5' and 3' of the CCC hotspot, i.e. at the 5 nucleotide recognition sequence positioning the CCC hotspot at the center. Similar results were obtained on nucleotide analog interference mapping analysis, which is a UDG-based assay [26]. We assume that introduction of 2'-OH caused a direct steric clash between the hydroxyl group and the surface of the catalytic pocket of A3G CD2 and/or an indirect one caused by conversion of the preferred sugar ring puckering from the C2'-endo to the C3'-endo conformation. A direct and/or indirect clash is supposed to prevent C6 from properly fitting into the catalytic pocket. A similar idea was proposed for AID to interpret the reduction of activity caused by 2'-F substitution in substrate DNA [41].

### The effect of 2′-OH substitution or abasic substitution in the flanking regions of a 5 nucleotide recognition sequence on deaminase activity

In the previous section we showed that a 5 nucleotide recognition sequence, containing a CCC hotspot at its center, is important for A3G CD2 to exert its full deaminase activity. Here, we attempted to determine whether or not the regions flanking the 5' and 3' to this 5 nucleotide recognition sequence play any role in the reactivity of the substrates. A series of 10-mer DNA oligonucleotides, each carrying either a ribonucleotide or abasic deoxyribonucleotide in the 5′- and 3′-flanking regions, was subjected to real-time monitoring.

**Fig 2:**
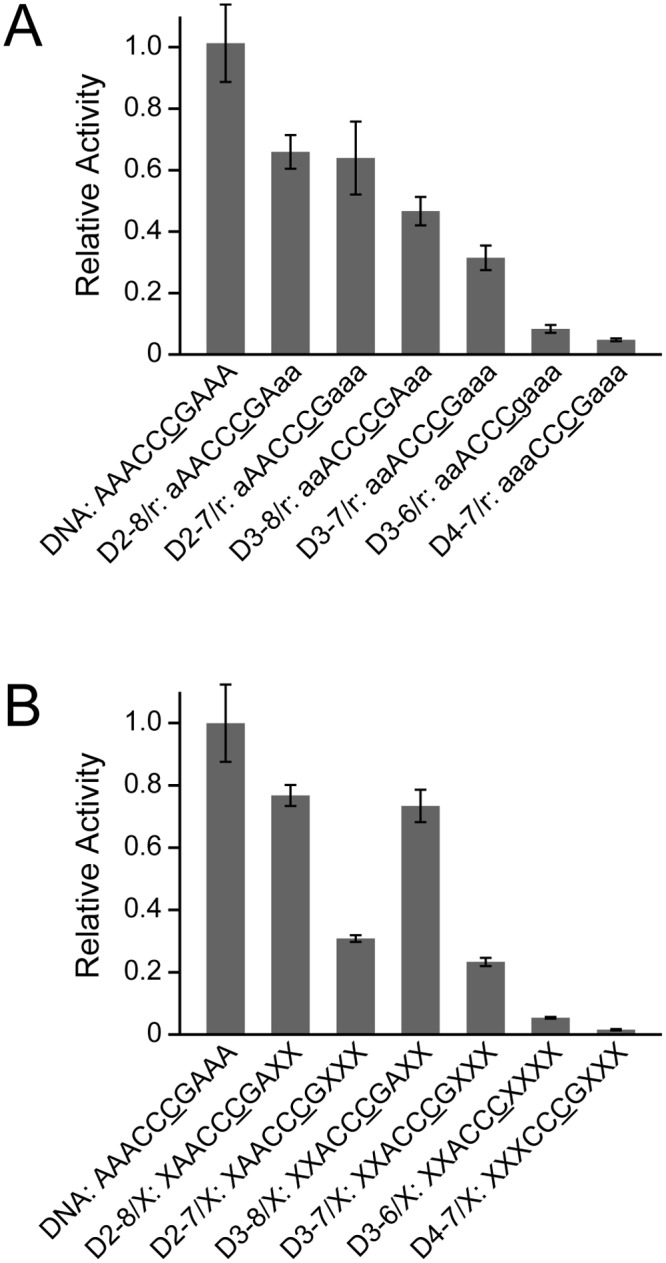
Real-time monitoring of deamination reactions for substrates containing either multiple ribonucleotides or abasic deoxyribonucleotides. Relative activity was calculated by dividing the deamination rate constant for a substrate containing either a ribonucleotide substitution **(A)** or an abasic deoxyribonucleotide substitution **(B)** by that for all DNA substrates. "a", "g", and "X" correspond to adenine and guanine ribonucleotides, and an abasic deoxyribonucleotide, respectively.

As shown in Fig 2A, the relative activities of A3G CD2 toward ssDNA substrates with multiple ribonucleotide substitutions were quantified. s1_D3-6/r, residues 3–6 being deoxyribonucleotides and the other residues being ribonucleotides, has a ribonucleotide substitution at residue G7 that is one of the 5 nucleotide recognition sequence and was indicated to be critical for deamination in the previous section. s1_D4-7/r (Table 1) also has a substitution in the 5 nucleotide recognition sequence at residue A3. The activity toward these substrates was extremely low (Fig 2A), which is consistent with the results in Fig 1C. Surprisingly, although the deoxyribonucleotides are retained in the 5 nucleotide recognition sequence and ribonucleotide substitutions are introduced only outside of the 5 nucleotide recognition sequence, the reactivity of the substrate s1_D3-7/r turned out to be rather low. This indicates that the sugar moiety of the nucleotides in the regions outside the 5 nucleotide recognition sequence also affects the deamination by A3G CD2, which was not apparent in the single substitution experiments shown in Fig 1. It is supposed that the effect of a substitution outside the 5 nucleotide recognition sequence on the activity is small but that the effect becomes evident when such a substitution is introduced at multiple positions. The substrates s1_D2-7/r and s1_D3-8/r each retain deoxyribonucleotides in the 5 nucleotide recognition sequence, and additionally at the 5′- and 3′- flanking positions of the 5 nucleotide recognition sequence, respectively. The reactivity of s1_D2-7/r was higher than that of s1_D3-8/r, which indicates that the introduction of a 2'-OH substitution at the 5'-flanking position of the 5 nucleotide recognition sequence has a higher negative effect on the deamination activity than one at the 3'-flanking position. Thus, the requirement of being a deoxyribonucleotide is higher at the 5'-flanking position than at the 3′-flanking position of the 5 nucleotide recognition sequence.

The relative activities of A3G CD2 toward ssDNA substrates that contain multiple abasic deoxyribonucleotides in the 5′- and 3′-flanking regions were quantified, as shown in Fig 2B. The substrates s1_D3-6/X and s1_D4-7/X exhibited extremely low reactivity (Fig 2B). This indicates that not only the sugar moiety but also the base moiety of the 5 nucleotide recognition sequence is critical for the reactivity. The substrate s1_D3-7/X retains deoxyribonucleotides in the 5 nucleotide recognition sequence and abasic substitutions are introduced outside of the 5 nucleotide recognition sequence. This substrate also showed a great reduction of reactivity, which indicates that the base moiety of the nucleotides in the regions outside the 5 nucleotide recognition sequence also affects the deamination reaction of A3G CD2. Each of the substrates s1_D2-7/X and s1_D3-8/X retains bases in the 5 nucleotide recognition sequence, and additionally at the 5′- and 3′- flanking positions of the 5 nucleotide recognition sequence, respectively. The reactivity of s1_D2-7/X was lower than that of s1_D3-8/X and was as low as that of s1_D3-7/X, which indicates that an abasic substitution at the 3′-flanking position of the 5 nucleotide recognition sequence has a higher negative effect on the deamination activity of A3G CD2 than one at the 3′-flanking position. Thus, the base moiety is more highly required at the 3′-flanking position than at the 5′-flanking position of the 5 nucleotide recognition sequence. Altogether, the substitution of the chemical moieties of nucleotides revealed that the regions relevant to the activity of A3G CD2 extends outside the 5 nucleotide recognition sequence defined in the previous section, and that a deoxyribose and a base are required at the 5'- and 3'-flanking positions of the 5 nucleotide recognition sequence, respectively.

### The effect of 2'-OH substitution or abasic substitution at the linker region connecting two CCC hotspots on the 3′→5′ polarity

In the former study, we used an ssDNA substrate containing two CCC hotspots, connected by a long linker region, to monitor the deamination reaction, and revealed that A3G CD2 can deaminate the two hotspots with 3′→5′ polarity [28]. It was also revealed that the 3′→5′ polarity is caused by sliding, because the polarity disappeared when sliding was inhibited through the formation of a DNA duplex at a linker region [28]. Here, we substituted the linker region of an ssDNA substrate with a poly-ribonucleotide or poly-abasic deoxyribonucleotide and then subjected it to real-time monitoring. Thus, we attempted to reveal the effects of these substitutions on the 3′→5′ polarity of A3G CD2's deamination activity.

**Fig 3:**
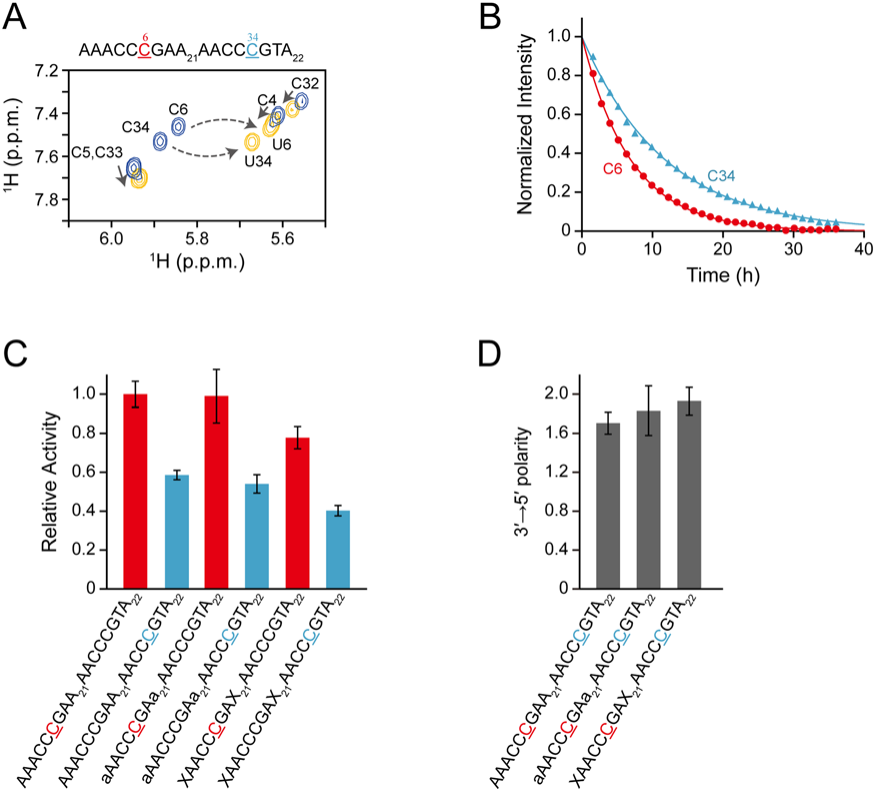
Real-time monitoring of deamination reactions for DNA oligonucleotides having two CCC hotspots linked by either a deoxyribonucleotide, ribonucleotide, or abasic deoxyribonucleotide. **(A)** TOCSY spectra of AAACCCGAA_21_AACCCGTA_22_ (C to U conversion occurs at underscored C6 and C34) recorded before (blue) and 36 hours (yellow) after addition of A3G CD2 are superimposed. Signals of C6 and C34 are each connected with those of U6 and U34 by a broken arrow. Note that the signals of C4, C5, C32, and C33 are also perturbed (solid arrows) due to the aforementioned C to U conversion. **(B)** The time course of the intensity change was monitored for C6 and C34 of the ssDNA shown in (A). **(C)** Relative activities toward C6 and C34 of AAACCCGAA_21_AACCCGTA_22_, aAACCCGAa_21_AACCCGTA_22_, and XAACCCGAX_21_AACCCGTA_22_ (C6 and C34 are underscored), where "a" and "X" are an adenine ribonucleotide and an abasic deoxyribonucleotide, respectively. Relative activity was defined as the ratio of the rate constant obtained for C6 or C34 of each substrate to that obtained for C6 of AAACCCGAA_21_AACCCGTA_22_. **(D)** The 3′→5′ polarity of A3G's deamination activity is defined as the ratio of the rate constant obtained for C6 to that obtained for C34 for each substrate.

Fig 3A shows an example of real-time monitoring with a standard 58-mer ssDNA substrate, containing two hotspots (s2_DNA). Presented in blue are the H5–H6 TOCSY correlation signals of the residues within the 5′-CCC hotspot (C4, C5, and C6) and 3'-hotspot (C33, C34, and C35) before the addition of A3G CD2. Upon C to U conversion at positions C6 and C34, all the signals were perturbed. The signals at the 36 hours time point are shown as an example of the perturbation (yellow). It can be seen clearly that the signals of C6 and C34 are separate from other signals, and therefore changes in intensity can be followed unambiguously. Thus, we plotted the normalized intensity against time for these signals (Fig 3B). We performed the same analysis with two substrates each having a poly-ribonucleotide (s2_RNA) and poly-abasic deoxyribonucleotide (s2_Abasic) as a linker. The rate constant was calculated for each substrate and the relative activity was obtained (Fig 3C).

The values for the relative activity of A3G CD2 toward C6 (in 5'-CCC hotspot) are almost the same for s2_DNA, s2_RNA, and s2_Abasic. Similarly, the values for the relative activity of A3G CD2 toward C34 (in 3'-CCC hotspot) are also almost the same for s2_DNA, s2_RNA, and s2_Abasic. Using these values, the 3′→5′ polarity of A3G CD2’s deamination activity toward each substrate was quantified as the ratio of the rate constant obtained for C6 to that obtained for C34 (Fig 3D). The values for the 3′→5′ polarity for s2_RNA and s2_Abasic are almost the same as that for s2_DNA. This finding indicates that either a 2′-OH substitution or abasic substitution in the linker region connecting two CCC hotspots does not have a negative effect on the 3′→5′ polarity of A3G CD2’s deamination activity. Since the chemical moiety that is conserved in these three substrates is the phosphate backbone, we hypothesized that the sugar and base moieties are not as important as the phosphate backbone for A3G CD2 to slide along ssDNA. In other words, we assume that the phosphate backbone tethers A3G CD2 to ssDNA via electrostatic interaction when A3G CD2 slides on an ssDNA.

### NaCl concentration dependence of the 3′→5′ polarity

**Fig 4:**
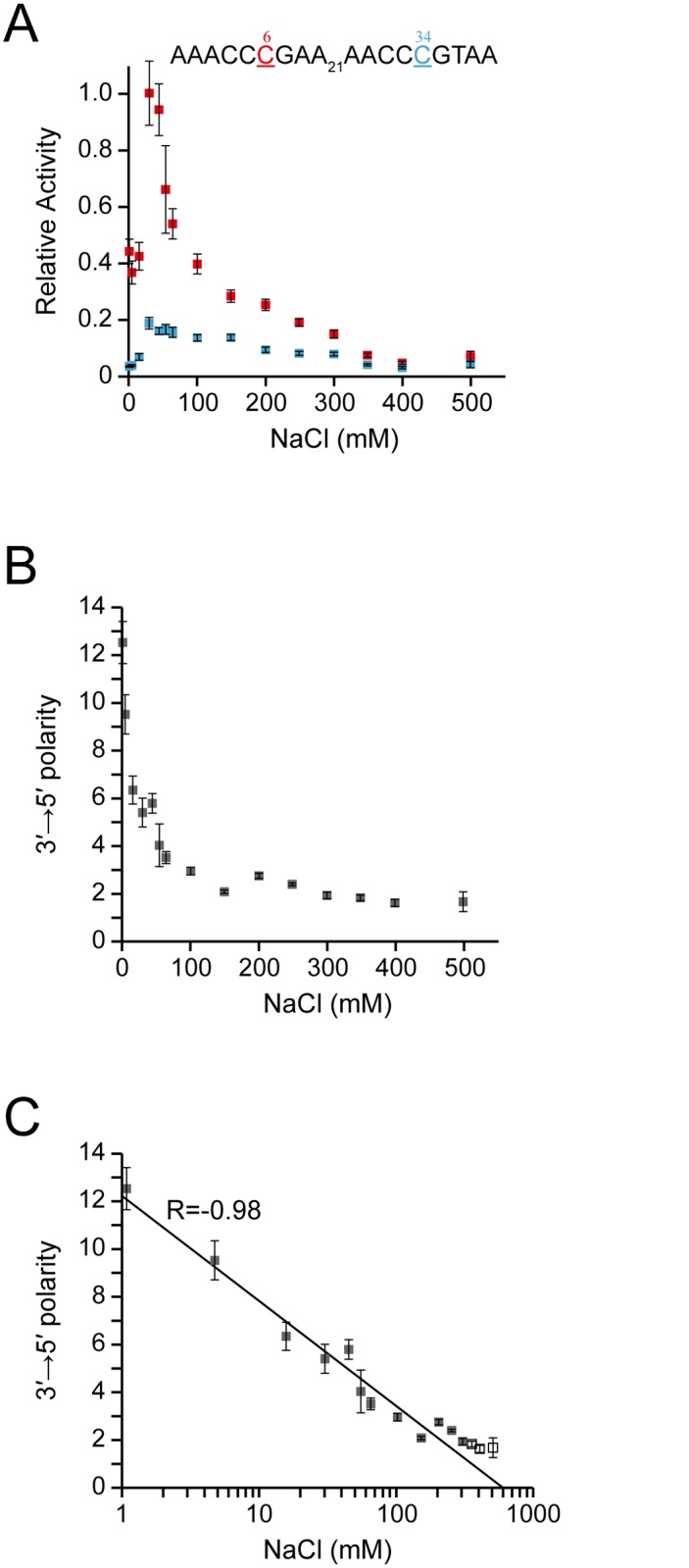
The effect of the NaCl concentration on the 3′→5′ polarity of A3G's deamination activity. **(A)** Relative activity was defined as the ratio of the rate constant obtained for C6 or C34 of AAACCCGAA_21_AACCCGTAA at each NaCl concentration to that obtained for C6 of AAACCCGAA_21_AACCCGTAA at 30 mM NaCl. **(B)** The 3′→5′ polarity of A3G's deamination activity is defined as the ratio of the rate constant obtained for C6 to that obtained for C34 at each NaCl concentration. **(C)** Plot of the 3′→5′ polarity against the logarithm of the NaCl concentration.

Our data suggested that the phosphate backbone of ssDNA contributes to its sliding. To confirm this, we analyzed the effect of the NaCl concentration on the 3′→5′ polarity of A3G CD2’s deamination activity. In Fig 4A, the values for the relative activity of A3G CD2 as to positions C6 and C34 (underlined) of the s3_DNA substrate (AAACCCGAA_21_AACCCGTAA) are plotted against NaCl concentrations. The relative activity was calculated as the ratio of the rate constant obtained for C6 or C34 of s3_DNA at each NaCl concentration to that obtained for C6 s3_DNA at 30 mM NaCl. The relative activity of A3G CD2 at both C6 and C34 was the highest at 30 mM NaCl. As the concentration of NaCl increased from 30 mM, the relative activity of A3G CD2 decreased. This is due to destabilization of the complex of A3G CD2 with the hotspot through a decrease of the electrostatic interaction. The decreased electrostatic interaction also causes the abortion of sliding, which results in a decrease of activity. The relative activity of A3G CD2 also decreased as the NaCl concentration decreased from 30 mM. In this case, the complex of A3G CD2 with the hotspot is stabilized excessively under low NaCl conditions, which could result in interference with turnover. Additionally, sliding slows down on tight interaction of A3G CD2 with the DNA strand, which results in a reduction of activity.

In Fig 4B, the values for the 3′→5′ polarity of the deaminase activity of A3G CD2 are plotted against NaCl concentrations. Interestingly, the value for the 3′→5′ polarity was highest at the lowest NaCl concentration investigated in the present study (1 mM). The value for the 3′→5′ polarity decreased rapidly as the concentration of NaCl increased, and eventually reached the value of approximately one around the concentration of 400 mM. This indicates that the efficiencies of deamination at the C6 and C34 sites became the same, namely, the 3′→5′ polarity was lost, which was probably due to the alleviation of sliding through facilitated dissociation of A3G CD2 from the substrate ssDNA. The value for the 3′→5′ polarity increased rapidly as the concentration of NaCl decreased. It is assumed that the sliding speed itself decreased at low NaCl concentrations due to tight electrostatic interaction of A3G CD2 with the DNA strand. However, it is also assumed that A3G CD2 hardly dissociated from the DNA strand and continued to slide along it, which resulted in the high 3′→5′ polarity value.

Interestingly, linear correlation was found between the 3′→5′ polarity and the logarithm of the NaCl concentration, the correlation coefficient being -0.98 (Fig 4C). The physico-chemical background to this correlation is not clear at this moment.

### Reactivity of A3G CD2 toward a substrate containing *5mC* or *5hmC*

Previously, Wijesinghe *et al*. showed that A3G exhibits no detectable deamination activity toward *5mC* using an *in vivo* method, the so-called Kanamycin-resistance reversion assay, which is a genetically modified *E*. *coli*-based method [38]. Carpenter *et al*. used DNA glycosylase-based methods to investigate the activity for *5mC* to T conversions by A3A and A3G prepared from human cells [37]. It was shown that although A3A can deaminate *5mC*, A3G cannot deaminate *5mC* within the experimental time (120 min). Here, we applied our real-time NMR method to detect, if any, the deamination activity of A3G CD2 toward *5mC* or *5hmC*. Because the real-time NMR method monitors the perturbation of the signal of *5mC* or *5hmC* itself, we expected to observe any low activity.

**Fig 5:**
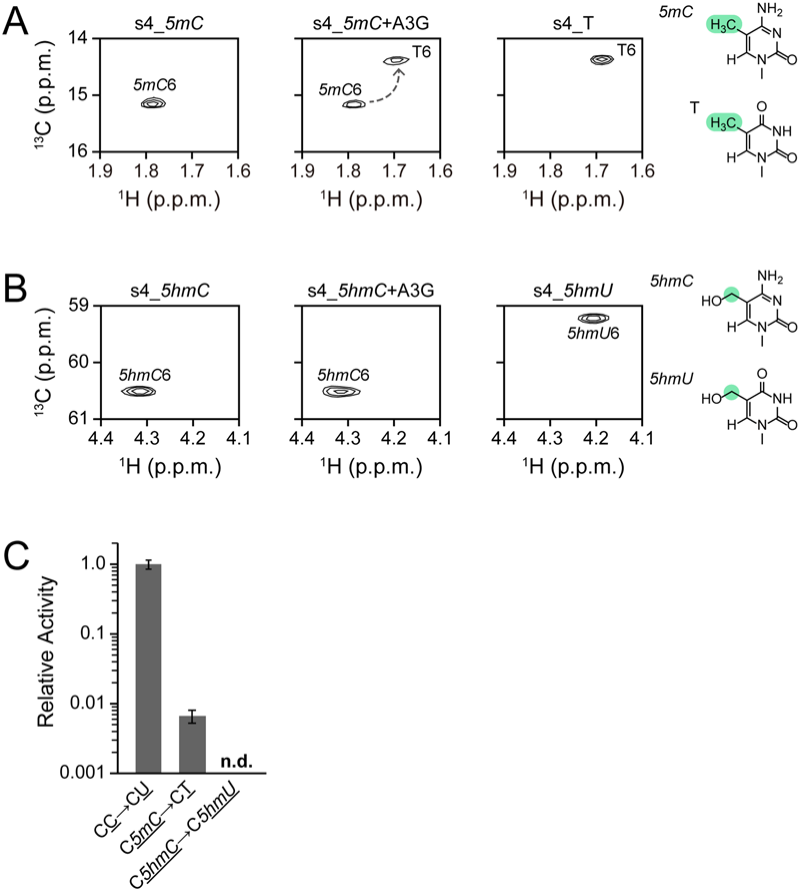
Real-time monitoring of deamination reactions of substrates containing 5-methylcytidine or 5-hydroxymethylcytidine at the target position. **(A)** ^1^H-^13^C HSQC spectrum of an ssDNA substrate, AAAAC*5mC*GAAA (left), and that recorded 1,900 hours after addition of A3G CD2 (center). The ^1^H-^13^C HSQC spectrum of AAAACTGAAA is shown as a reference (right). **(B, top)** ^1^H-^13^C HSQC spectrum of an ssDNA substrate, AAAAC*5hmC*GAAA (left), and that recorded 1,700 hours after addition of A3G CD2 (center). The ^1^H-^13^C HSQC spectrum of AAAAC*5hmU*GAAA is shown as a reference (right). **(C)** Comparison of the relative activity between the reactions of three substrates, AAAACCGAAA, AAAAC*5mC*GAAA, and AAAAC*5hmC*GAAA.

We used 5'-AAAAC*5mC*GAAA-3' (s4_*5mC*) as a substrate, whose standard sequence is 5'-AAAACCGAAA-3' (s4_C) and which contains two cytidines, one of which is the target (underlined). Fig 5A presents the ^1^H-^13^C HSQC spectrum for the methyl groups of *5mC* and thymidine, in which the *5mC*6 and T6 signals are labeled, respectively. The spectrum at 1,900 hours after the addition of A3G CD2 (Fig 5A, middle panel) clearly shows that *5mC* of s4_*5mC* is converted to thymidine to some extent, since a T6 signal is present, as seen for a control DNA, s4_T (Fig 5A, right panel). This is the first time to detect the deamination activity of A3G toward *5mC*. It is assumed difficult for the *in vivo* method to detect such weak activity.

Next, we investigated the reactivity of substrates containing *5hmC* for the deaminase reaction. 5'-AAAAC*5hmC*GAAA-3' (s4_*5hmC*) was subjected to real-time monitoring (Fig 5B). The ^1^H-^13^C HSQC spectra for the methylene groups of *5hmC* and 5-hydroxymethyluridine (*5hmU*) are presented in Fig 5. The spectrum at 1,700 hours after the addition of A3G CD2 (Fig 5B, middle panel) was exactly the same as that at time zero (Fig 5B, left panel), which is different from that of a control DNA, s4_*5hmU* (Fig 5B, right panel). The measurement was continued as long as 3,000 hours, however, no change in the spectral pattern was observed. Thus, our results explicitly showed that A3G has failed to deaminate *5hmC*.

Fig 5C compares the relative activity values for s4_C, s4_*5mC*, and s4_*5hmC*. The difference in structure between cytidine, *5mC*, and *5hmC* is the steric bulkiness of the base moiety, which increases in that order. Nabel *et al*. synthesized a series of substrates with unnatural C5 substituents of varied steric and electronic character, and investigated the reactivity of human AID and mouse APOBECs (1, 2, and 3) by means of a DNA glycosylase-based assay [36]. They concluded that deamination decreases with increasing steric bulkiness at the 5 position of cytidine. We assume that this is also the case for A3G, in other words, A3G's activity also decreases with increasing steric bulkiness at the 5 position of cytidine.

## Conclusion

In this study, we investigated the nucleic acid determinants for deamination by A3G CD2 using a real-time NMR method in combination with a series of ssDNA substrates each carrying a nucleotide analog at a single or multiple positions. Firstly, we showed that the sugar and base moieties of the consecutive 5 nucleotides, positioning the CCC hotspot at the center, as well as the 5'- and 3'-flanking regions of the consecutive 5 nucleotides, play an important role for A3G CD2 to exert its deamination activity. Secondly, we showed that A3G CD2 can tolerate the introduction of either a 2'-OH substitution in the sugar moiety or the removal of the base in the consecutive poly-nucleotide to slide along an ssDNA and exert the 3′→5′ polarity. This suggests that the electrostatic interaction between A3G CD2 and the phosphate backbone of an ssDNA is the key for sliding. This idea was confirmed by the fact that the 3′→5′ polarity is dependent on the NaCl concentration. Thirdly, we attempted to detect the activity of A3G CD2 toward an ssDNA containing *5mC* or *5hmC* at the targeted cytidine site by direct observation. We showed for the first time that A3G CD2 can convert *5mC* to thymidine, although it cannot convert *5hmC* to *5hmU*. Altogether we have observed A3G CD2's deamination reaction directly and unambiguously, and thus we have successfully showed that the combination of a real-time NMR method and a chemically modified nucleotide substrates is a powerful strategy for analyzing deamination activity. In the future, if the preparation of the full-length A3G becomes easily accessible for NMR study, our current analysis can be applied. It would be interesting to compare the data obtained for the full-length A3G and those obtained for A3G CD2 in this study.
